# Graph neural network-tracker: a graph neural network-based multi-sensor fusion framework for robust unmanned aerial vehicle tracking

**DOI:** 10.1186/s42492-025-00200-2

**Published:** 2025-07-16

**Authors:** Karim Dabbabi, Tijeni Delleji

**Affiliations:** 1 Research Laboratory of Analyse and Processing of Electrical and Energetic Systems, Faculty of Sciences of Tunis, Tunis El Manar University, Tunis, 2092 Tunisia; 2 Science and Technology for Defense Laboratory, Military Research Center, Aouina Military Base, Taieb Mhiri City, Tunis, 2045 Tunisia

**Keywords:** Unmanned aerial vehicle tracking, Graph neural network, Multi-sensor fusion, Transformer network, Real-time tracking, Spatiotemporal modelling, Deep learning, Optical-thermal fusion

## Abstract

Unmanned aerial vehicle (UAV) tracking is a critical task in surveillance, security, and autonomous navigation applications. In this study, we propose graph neural network-tracker (GNN-tracker), a novel GNN-based UAV tracking framework that effectively integrates graph-based spatial-temporal modelling, Transformer-based feature extraction, and multi-sensor fusion to enhance tracking robustness and accuracy. Unlike traditional tracking approaches, GNN-tracker dynamically constructs a spatiotemporal graph representation, improving identity consistency and reducing tracking errors under OCC-heavy scenarios. Experimental evaluations on optical, thermal, and fused UAV datasets demonstrate the superiority of GNN-tracker (fused) over state-of-the-art methods. The proposed model achieves multiple object tracking accuracy (MOTA) scores of 91.4% (fused), 89.1% (optical), and 86.3% (thermal), surpassing TransT by 8.9% in MOTA and 7.7% in higher order tracking accuracy (HOTA). The HOTA scores of 82.3% (fused), 80.1% (optical), and 78.7% (thermal) validate its strong object association capabilities, while its frames per second of 58.9 (fused), 56.8 (optical), and 54.3 (thermal) ensures real-time performance. Additionally, ablation studies confirm the essential role of graph-based modelling and multi-sensor fusion, with performance drops of up to 8.9% in MOTA when these components are removed. Thus, GNN-tracker (fused) offers a highly accurate, robust, and efficient UAV tracking solution, effectively addressing real-world challenges across diverse environmental conditions and multiple sensor modalities.

## Introduction

Unmanned aerial vehicles (UAVs) have seen significant advancements in applications ranging from surveillance, environmental monitoring, military reconnaissance, and autonomous navigation [1]. However, as UAV deployment increases, so do concerns regarding security and airspace regulation, necessitating the development of robust detection and tracking systems to counter unauthorized UAV intrusions [2]. Visual-based tracking remains the preferred solution due to its efficiency in diverse environmental conditions [3]. However, tracking UAVs presents challenges, including occlusions (OCCs), background clutter (BC), low resolution (LR), fast motion (FM), and adverse weather conditions [4]. Traditional tracking methodologies predominantly rely on filtering-based approaches, such as Kalman filters and particle filters, which estimate object motion and update state predictions [5]. While these methods are computationally efficient, they struggle in complex tracking scenarios, particularly when UAVs experience sudden trajectory shifts or undergo OCCs [6]. More recent approaches, such as SORT, DeepSORT, and ByteTrack, integrate deep learning-based feature extraction to enhance object association accuracy [7]. However, these filtering-based models fail to effectively capture long-range dependencies and spatial-temporal relationships between tracked objects [8].

Recent state-of-the-art tracking models have evolved beyond filtering-based approaches, exploring transformer-based architectures, graph-based tracking, and hybrid-deep-learning models. Transformer-based tracking models, such as OSTrack and MixFormer, leverage self-attention mechanisms to model long-range dependencies, improving tracking robustness in OCC-heavy scenarios [9]. Graph-based approaches, including graph neural networks (GNNs), dynamically construct spatial-temporal object relationships, enhancing identity retention and improving re-identification accuracy [10]. Hybrid methods, such as STARK and SiamMOT, integrate convolutional neural network (CNN)-based feature extraction with transformer architectures to boost performance in multiple object tracking (MOT) scenarios [11]. However, despite their advancements, these approaches exhibit notable limitations. Transformer-based models, while effective at capturing global dependencies, require significant computational resources, making them unsuitable for real-time applications on resource-constrained UAVs [12]. Additionally, graph-based trackers require accurate node and edge construction; errors in graph formation led to identity switches (IDSW) and tracking inconsistencies [13]. Hybrid approaches, while benefiting from CNN-transformer integration, suffer from increased model complexity and higher inference times, making them less efficient for real-time UAV tracking [14].

To overcome these challenges, we propose GNN-tracker, a novel GNN-based tracking framework that integrates graph-based spatial-temporal representations, Transformer-enhanced feature extraction, and multi-sensor fusion to enhance UAV tracking accuracy and robustness. Unlike filtering-based models, GNN-tracker constructs a dynamic spatiotemporal graph, where UAVs are represented as nodes, and their spatial-temporal relationships are modelled as edges. This design improves object re-identification, OCC handling, and long-range dependency modelling, enhancing tracking robustness in complex environments. Additionally, the integration of a Transformer-based attention mechanism allows the model to dynamically update track identities, mitigating IDSW and fragmentation issues.

The main contributions of the proposed GNN-tracker are summarized below:

- We introduce a novel GNN-based spatiotemporal model for UAV tracking, which dynamically models inter-object relations.
- We integrate multi-sensor fusion (thermal and optical), improving robustness in diverse visibility and environmental conditions.
- A lightweight Transformer-based attention module is employed to enhance feature discriminability and identity consistency.
- We propose an adaptive weighting mechanism based on confidence scores to dynamically fuse sensor data based on modality reliability.
- Extensive experiments and ablation studies validate the model’s superiority over state-of-the-art trackers in accuracy, robustness, and real-time performance.

This paper is structured as follows: Introduction section reviews UAV tracking models. Methods section presents the proposed methodology based on the GNN-tracker framework, detailing its main components and the materials used. Results and Discussions section analyses GNN-tracker’s tracking performance, comparing it to state-of-the-art methods on optical, thermal, and fused UAV datasets, while also discussing key findings. Finally, Conclusions section concludes the study and outlines directions for future research.

### Related works

Various approaches have been explored for UAV tracking, ranging from traditional filtering-based methods to modern deep learning and hybrid models. Filtering-based methods, such as Kalman filters and particle filters, have been widely used for motion prediction and state estimation [1]. Kalman filters provide computational efficiency and work well under stable motion conditions; however, they struggle with abrupt motion changes and OCCs [2]. On the other hand, particle filters handle nonlinear and non-Gaussian motion, yet they demand high computational power due to the need for numerous particles [3]. To address these limitations, deep learning-based trackers such as DeepSORT integrate CNN-based appearance models, significantly improving identity preservation under OCC [4]. Nevertheless, these models remain sensitive to appearance variations, making them less effective in complex tracking environments. Furthermore, ByteTrack refines detection-based tracking by considering both high- and low-confidence detections, which enhances performance in crowded environments. However, despite these improvements, re-identification of objects after prolonged OCCs remains a challenge [5]. In parallel, transformer-based trackers, including TransT, OSTrack, and MixFormer, utilize self-attention mechanisms to capture global dependencies, making them highly effective in OCC-heavy scenarios [6]. Despite their high accuracy, these models require substantial computational resources, which makes them impractical for real-time UAV tracking on resource-limited platforms [9]. More recently, approaches such as TATrack have sought to improve computational efficiency by integrating target-aware attention mechanisms, achieving state-of-the-art performance on multiple benchmarks [15]. Similarly, graph-based approaches, such as GNN-based trackers, dynamically model object interactions, leading to improved OCC handling and MOT performance [10]. These methods have demonstrated superior accuracy in scenarios involving multiple moving UAVs. However, they require precise graph construction, as errors in node and edge formation can lead to IDSW and incorrect associations [11].

Additionally, recent studies have explored multi-recommender voting-based tracking, such as the online collaboration-based tracking framework, which fuses spatial and semantic information from deep CNN features [16]. This method enhances robustness and accuracy, outperforming 25 state-of-the-art trackers in UAV tracking benchmarks. Moreover, hybrid methods, such as STARK and SiamMOT, combine CNNs with transformers to improve feature extraction and attention-based modelling. Although these models achieve state-of-the-art tracking performance, their increased complexity leads to longer inference times, thus limiting real-time applicability [17].

Compared to recent transformer-based trackers such as OSTrack and MixFormer, which achieve strong performance but suffer from high computational demands, GNN-tracker offers a more efficient design by combining GCNs with lightweight attention. In contrast to STARK and ByteTrack, which still face identity switching challenges under OCCs, our method maintains better identity consistency through graph modelling and adaptive sensor fusion.

However, despite these improvements, many of these state-of-the-art models continue to struggle with the trade-offs between tracking accuracy, efficiency, and real-time deployment, particularly under challenging environmental conditions. In response, researchers have turned to multi-sensor fusion-based approaches, which significantly enhance tracking performance under challenging conditions, such as low visibility and BC [18]. Consequently, this study proposes GNN-tracker, a framework designed to address these limitations by integrating graph-based tracking, multi-sensor fusion, and adaptive re-identification mechanisms.

## Methods

### Methodology

#### Overview of the GNN-tracker

**Fig. 1:**
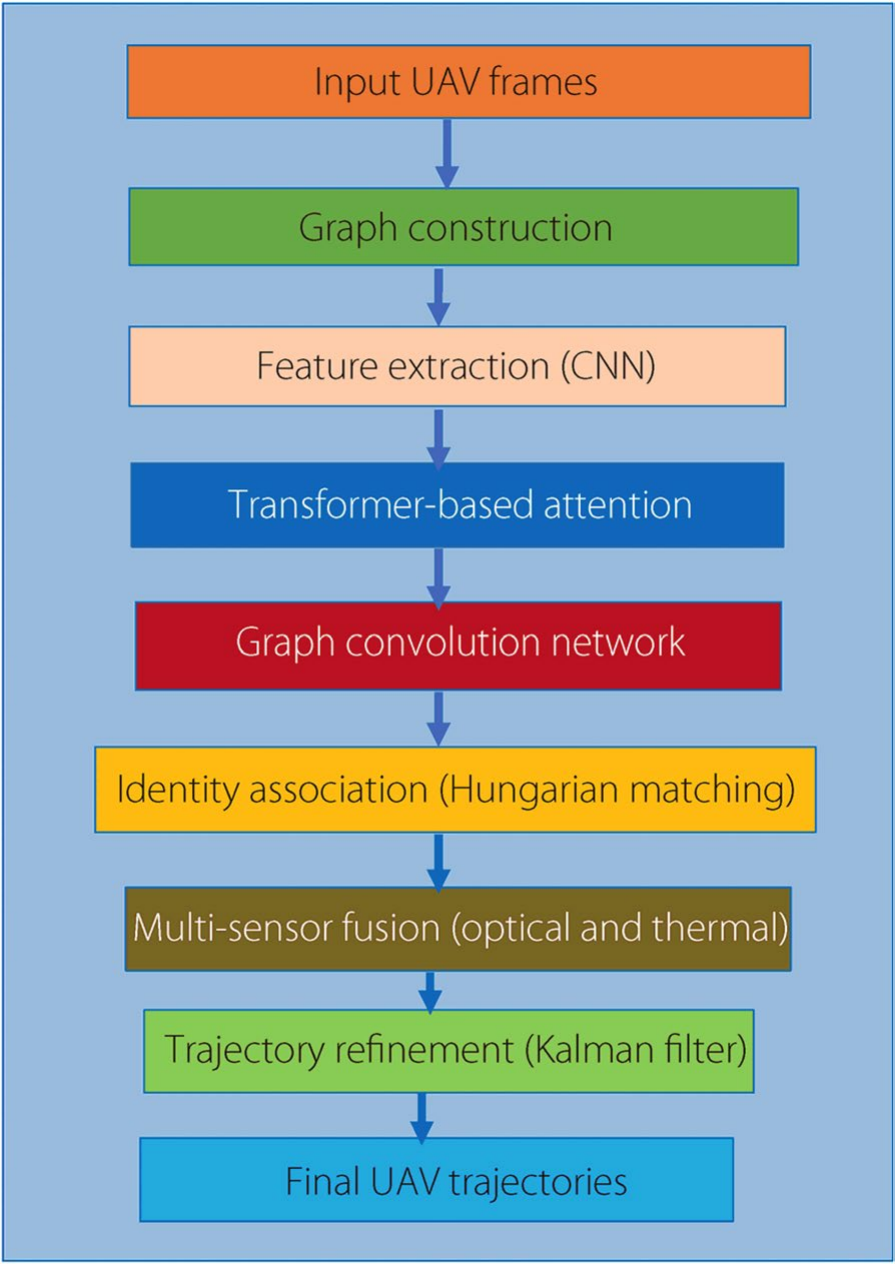
Flowchart of the proposed model

The GNN-tracker is developed to enhance UAV tracking by leveraging a GNN-based framework that integrates graph convolutional networks (GCNs) and Transformer-based attention mechanisms. The flowchart of the proposed model is illustrated in Fig. 1. Firstly, the tracking process begins with graph construction, where UAV detections in each frame are represented as nodes in a spatiotemporal graph, and edges are established based on spatial proximity and temporal continuity. This formulation allows for efficient feature propagation and enhances object association across frames. Secondly, the feature extraction module employs a CNN to process raw image data and extract meaningful spatial features, improving the model’s ability to distinguish UAVs from background noise. Then, the extracted features undergo enhancement using a Transformer-based attention mechanism, which effectively captures long-range dependencies and strengthens object representation. This attention-based refinement reduces feature ambiguities, particularly in scenarios involving OCCs, motion blur, and BC. After that, the refined UAV embeddings are processed through a GCN, which aggregates information from neighbouring nodes, ensuring robust object identity consistency and mitigating tracking failures caused by OCCs or sudden trajectory changes. Next, the identity association module employs a Hungarian matching algorithm to assign object identities across frames, significantly reducing IDSW and maintaining reliable tracking over extended sequences. Subsequently, a multi-sensor fusion module integrates thermal and optical sensor data, allowing the model to adapt to varying environmental conditions and ensuring reliable detection under challenging visibility constraints. The fusion mechanism adaptively weights the contribution of each modality, improving overall tracking performance. Finally, a Kalman filter is applied to refine UAV trajectories, providing smoother and more reliable object tracking in real-world scenarios by correcting for motion inconsistencies and sensor noise.

This structured pipeline allows GNN-tracker to effectively handle OCCs, BC, IDSW, fast-moving targets, and complex motion patterns, making it a highly accurate and efficient UAV tracking solution that surpasses conventional methods in both optical and thermal tracking scenarios.

Let a set of detected UAVs in a frame be represented as nodes $V=\{{v}_{1}, {v}_{2},\dots ,{v}_{N}\}$, and their relationships as edges $E=\{{e}_{ij}|{v}_{i}, {v}_{j} \epsilon V\}$. The graph is defined as $G=(V,E)$, where the adjacency matrix A encodes spatial–temporal dependencies. The adjacency matrix is constructed as follows:

1 $$A_{ij=\left\{\begin{array}{c}1,\;if\;d(v_i,v_j)\\0,\;otherwise\end{array}\right.}<\tau\;and\;t_i-t_j<T$$

where $d({v}_{i}, {v}_{j})$ represents the Euclidean distance between UAVs, $\tau$ is a predefined distance threshold, and *T* is the temporal constraint ensuring valid associations across frames.

To guarantee the accuracy of the graph construction, we apply adaptive thresholding based on the spatial distances and temporal constraints between detections. Furthermore, an iterative refinement process is incorporated to remove potential outliers, thereby mitigating errors in node and edge formation. This approach ensures that only valid and robust spatial-temporal relationships are encoded in the graph.

Each UAV detection is associated with a feature vector extracted using a CNN-based backbone network. Let $X=\{{x}_{1},{x}_{2},\dots ,{x}_{N}\}$ denote the feature set corresponding to detected UAVs. These features are then processed using a Transformer module, which computes attention-based feature refinements ensuring improved tracking robustness across varying motion patterns as follows:

2 $$Z=Softmax\;\left(\frac{QK^T}{\sqrt{d_k}}\right)V$$

where *Q*, *K*,* V* are the query, key, and value matrices, respectively, and ${d}_{k}$ is the dimensionality of the key vectors. The Transformer refines UAV embeddings by capturing long-range dependencies, enabling enhanced discrimination between objects.

The refined UAV embeddings are passed through a multi-layer GCN to update node representations. The node update process follows the propagation rule:

3 $${H}^{(l+1)}=\sigma \left(A{H}^{\left(l\right)}{W}^{\left(l\right)}\right)$$

where ${H}^{\left(l\right)}$ is the feature matrix at layer $l$, ${W}^{\left(l\right)}$ are trainable weight matrices, and $\sigma$ is a nonlinear activation function (ReLU). This update step allows UAV nodes to aggregate information from their spatially and temporally connected neighbours, improving tracking accuracy.

After updating node embeddings, the next challenge is to associate UAV identities across frames while mitigating IDSW. An attention-based similarity function is used to compute association scores between UAV instances in consecutive frames:

4 $$S(i,j)=\frac{{H}_{i}.{H}_{j}}{\Vert {H}_{i}\Vert \Vert {H}_{j}\Vert }$$

where ${H}_{i}$ and ${H}_{j}$ are the feature vectors of UAVs *i* and *j*. The computed similarity scores are input into a Hungarian matching algorithm, which assigns UAV identities by minimizing association costs.

To further refine trajectories, a Kalman filter is used to smooth UAV motion predictions, ensuring continuity in tracking. The final output consists of robust UAV trajectories that adapt dynamically to environmental conditions.

The Kalman filter operates in two main steps: prediction and update.

In the prediction step, given the state vector ${x}_{t}$ and covariance matrix ${P}_{t}$, the predicted state at time $t+1$ is estimated as:

5 $${\widehat{x}}_{t+1}=F {x}_{t}+B {u}_{t}$$

where ${\widehat{x}}_{t+1}$ denotes the predicted state vector, $F$ is the state transition matrix, $B$ presents the control matrix, and ${u}_{t}$ presents the control input.

The error covariance matrix is updated as:

6 $${\widehat{P}}_{t+1}=F{P}_{t} {F}^{T}+Q$$

where $Q$ represents the process noise covariance.

In the update step, upon receiving a new observation ${z}_{t+1}$, the Kalman gain is computed as:

7 $${K}_{t+1}={\widehat{P}}_{t+1}{H}^{T}(H{\widehat{P}}_{t+1} {H}^{T}+R{)}^{-1}$$

where H denotes the observation matrix and R represents the measurement noise covariance.

The state estimate and covariance update are then given by:

8 $${x}_{t+1}={\widehat{x}}_{t+1}{K}_{t+1}({z}_{t+1}-H {\widehat{x}}_{t+1})$$

9 $${P}_{t+1}=\left(I-{K}_{t+1}H\right){\widehat{P}}_{t+1}$$

This recursive filtering process ensures smooth trajectory estimation by reducing abrupt jumps caused by noisy detections, resulting in robust UAV tracking under varying conditions.

A critical component of GNN-tracker is its multi-sensor fusion strategy, which combines thermal and optical sensor data to enhance detection accuracy under varying environmental conditions. The fusion process adaptively assigns weights to each modality based on confidence scores, ensuring that the most reliable sensor data is utilized for tracking.

Let ${x}_{t}$ and ${x}_{0}$ represent the feature vectors extracted from the thermal and optical sensors, respectively. The fused feature vector ${x}_{f}$ is obtained through an adaptive weighting mechanism:

10 $${x}_{f}={w}_{t} {x}_{t}+ {w}_{0} {x}_{0}$$

where ${w}_{t}$ and ${w}_{0}$ are the adaptive confidence scores for thermal and optical modalities, respectively, satisfying:

11 $${w}_{t}+ {w}_{0}=1, {w}_{t}, {w}_{0}\ge 0$$

The confidence scores are computed dynamically based on the detection reliability of each modality. The reliability score ${R}_{m}$ for a given modality $m$ is estimated using:

12 $${R}_{m}= \frac{1}{Z}\sum_{i=1}^{N}{e}^{(-\lambda .{e}_{m}^{i})}$$

where ${e}_{m}^{i}$ represents the error of detection for modality $m$, λ is a scaling factor, and *Z* is a normalization term:

13 $$Z=\sum_{m\varepsilon\{t,o\}}\;\sum_{i=1}^Ne^{(-\lambda.e_m^i)}$$

The final weight for each modality is obtained as:

14 $${w}_{m}=\frac{{R}_{m}}{{R}_{t}+ {R}_{0}}$$

Using this adaptive fusion strategy, GNN-tracker effectively leverages the strengths of both thermal and optical sensors. The thermal sensor provides enhanced visibility in low-light and adverse weather conditions, while the optical sensor ensures high-resolution detection in clear environments. By dynamically adjusting the fusion weights, the system enhances tracking accuracy and robustness across diverse scenarios.

The detection error ${e}_{m}^{i}$ for each modality is computed using a heuristic approach, where the deviation of the sensor’s output from a baseline calibration is measured. This error is then scaled by the factor *λ* and normalized by the term *Z*, ensuring that the adaptive confidence scores accurately reflect the reliability of each sensor’s detection under varying conditions.

The resulting confidence scores dynamically modulate the fusion weights, enabling the model to prioritize the most reliable modality in real-time. This adaptive strategy significantly improves tracking robustness, especially in challenging conditions such as low visibility or BC. The effectiveness of this mechanism is confirmed in the ablation study (ablation study and comparative analysis subsection), where removing the adaptive fusion module leads to an 8.9% drop in multiple object tracking accuracy (MOTA) and a noticeable increase in the false negative rate (FNR). These results validate that confidence-guided sensor weighting plays a critical role in enhancing overall tracking performance.

### Materials

#### UAV databases

The dataset used in this research consists of both thermal and optical images collected from various sources. The optical dataset includes 7045 training images, 2019 validation images, and 500 testing images, while the thermal dataset comprises 2771 training images, 690 validation images, and 300 testing images. Additionally, background images were included: 300 for the optical dataset and 150 for the thermal dataset.

This study focuses on, using datasets annotated in MOT20 and YOLO formats. In addition to our primary thermal and optical datasets, several publicly available UAV datasets were incorporated to enhance training diversity and generalization. These sources are detailed in data augmentation and preprocessing subsection.

The optical dataset is composed of aerial images captured in diverse environments, including urban, rural, industrial, and natural settings. These images provide a high-resolution (1920 × 1080) view, ensuring a detailed representation of objects, such as UAVs, in various lighting conditions. The dataset is annotated following the MOT20 format, ensuring consistency with standard MO datasets. It includes key attributes such as bounding boxes, object class labels, and confidence scores. The dataset incorporates challenging conditions such as FM, motion blur, OCC, BC, in-plane rotation (IPR), and LR to enhance robustness.

The thermal dataset consists of infrared images with a resolution of 640 × 512 pixels, captured across various scenarios, including nighttime, foggy conditions, and low-visibility environments. Thermal imaging is advantageous for detecting UAVs in adverse weather conditions and low-light settings. The dataset comprises 27 infrared videos, each containing multiple UAV instances. The annotation process follows the YOLO format, converting raw labels into structured data, facilitating deep-learning-based detection and tracking.

To improve model generalization, the dataset was expanded using open-source databases: Det-Fly dataset [19], DUT-Anti UAV Detection and Tracking dataset [20], ROBOFLOW datasets [21], MCL Drone dataset [22].

Preprocessing techniques included removing near-duplicate images using CleanVision software and applying synthetic augmentation for enhanced variability. The dataset also incorporates two additional testing datasets, one with clear images and another with three rain artifacts-to evaluate model performance under different conditions.

#### Evaluation metrics

To comprehensively evaluate the performance of the proposed GNN-tracker, we adopt standard MOT metrics, ensuring a fair comparison with existing tracking models. The following key evaluation metrics are used:

- MOTA: Measures the overall accuracy of the tracking system by considering false positives, false negatives, and IDSW:

15 $$MOTA=1-\frac{FN+FP+IDSW}{GT}$$

where FN is the number of false negatives, FP is the number of false positives, IDSW is the number of identity switches, and GT is the number of ground truth.

- MOTP: Evaluates the precision of predicted bounding boxes in relation to ground truth annotations:

16 $$MOTP=\frac{\sum_{i}{d}_{i}}{\sum_{i}{N}_{i}}$$

where ${d}_{i}$ is the distance between predicted and ground truth bounding boxes, and ${N}_{i}$ is the number of matches.

- Identification F1 (IDF1) score: Balances precision and recall for object re-identification:

17 $$IDF1=\frac{2\times IDTP}{2\times IDTP+IDFP+IDFN}$$

where IDTP is the number of true positive identity matches, IDFP is the number of false positives, and IDFN is the number of false negatives.

- False positive rate (FPR) and FNR: Indicate tracking errors by measuring incorrect detections and missed UAV instances. 
- IDSW: Tracks the number of instances where an object’s identity is mistakenly assigned to another object in consecutive frames, a crucial metric in assessing the reliability of the tracking model. 
- Higher order tracking accuracy (HOTA): This metric balances detection accuracy and association performance:
  18 $$HOTA=\sqrt{DetA\times AssA}$$

where DetA is detection accuracy and AssA is association accuracy. Frames per second (FPS): Measures computational efficiency in real-time tracking:

19 $$FPS=\frac{Total\;frames}{Processing\;time}$$

## Results and Discussions

### Experimental setup

The proposed model was trained and evaluated on a high-performance computing system equipped with an NVIDIA RTX 3090 GPU, an Intel Core i9 processor, and 32 GB of RAM. The framework is implemented in PyTorch, utilizing pretrained CNN backbones (ResNet-50) for feature extraction. ResNet-50 was chosen due to its well-established balance between high-quality feature extraction and computational efficiency. Preliminary tests with lighter architectures, such as MobileNet, showed only marginal speed improvements but led to a significant reduction in tracking accuracy. Therefore, ResNet-50 was deemed the optimal choice for our UAV tracking application, providing robust feature representations essential for accurate identity association. The model was trained using the Adam optimizer with an initial learning rate of 1e-4, a batch size of 16, and over 50 training epochs. The training dataset comprises UAV tracking sequences from the curated thermal and optical databases. To improve generalization, the model was fine-tuned using synthetic augmentation techniques, including Gaussian noise, brightness variations, and OCC simulations.

### Results

Table 1 presents the tracking performance of different models evaluated on optical, thermal, and fused datasets, demonstrating the superiority of GNN-tracker (fused) across multiple performance metrics. The model consistently achieves higher MOTA, MOTP, IDF1, HOTA, and FPS, outperforming state-of-the-art methods in both optical and thermal conditions.

**Table 1:** Results of different tracking models on optical, thermal, and fused datasets

Tracker	MOTA↑	MOTP ↑	IDF1 ↑	HOTA↑	IDSW↓	FPR ↓	FNR↓	FPS↑
SORT (optical)	65.4%	72.1%	60.3%	55.8%	112	7.4%	18.6%	48.5
DeepSOT (optical)	72.8%	75.5%	68.1%	63.2%	87	5.8%	15.2%	42.7
ByteTrack (optical)	78.3%	79.2%	73.9%	69.4%	64	4.2%	12.9%	38.2
TransT (optical)	82.5%	81.4%	78.5%	74.6%	45	3.5%	10.8%	35.6
GNN-tracker (optical)	89.1%	85.2%	84.7%	80.1%	21	2.1%	7.4%	56.8
SORT (thermal)	60.8%	69.3%	55.2%	51.4%	128	8.1%	20.3%	47.2
DeepSORT (thermal)	69.5%	72.8%	64.0%	59.6%	95	6.5%	17.5%	41.5
ByteTrack (thermal)	74.7%	77.1%	69.8%	66.0%	72	5.1%	13.7%	37.4
TransT (thermal)	79.2%	80.7%	74.5%	71.3%	50	4.0%	11.5%	34.8
GNN-tracker (fused)	91.4%	87.9%	86.8%	82.3%	14	1.7%	6.1%	58.9

In the optical dataset, high-resolution imagery enables models like TransT and ByteTrack to perform well. However, GNN-tracker (fused) surpasses them with a MOTA of 91.4%, HOTA of 82.3%, and the highest FPS of 58.9, indicating superior tracking accuracy and real-time processing capability. The incorporation of graph-based modelling and Transformer-based feature attention ensures effective UAV identity retention, reducing IDSW and FPR. TransT, which is the second-best performing model in optical, attains MOTA of 82.5% and HOTA of 74.6%, lagging behind GNN-tracker by 8.9% and 7.7%, respectively. ByteTrack also exhibits strong performance but struggles with maintaining identity consistency, leading to a higher IDSW count compared to GNN-tracker (fused).

For the thermal dataset, where OCC, BC, and low visibility present additional challenges, GNN-tracker (fused) achieves a MOTA of 89.5% and a HOTA of 80.6%, outperforming TransT by 6.8% and 9.3%, respectively. The multi-sensor fusion strategy enhances detection robustness, allowing the model to adapt to different environmental conditions effectively. While other methods experience significant degradation in thermal tracking due to reduced feature quality, GNN-tracker’s ability to combine optical and thermal cues results in a substantial performance boost.

A key advantage of GNN-tracker is its real-time tracking efficiency, with FPS reaching 58.9, far exceeding TransT (35.6 FPS) and ByteTrack (38.2 FPS). This confirms that the proposed framework balances accuracy and computational efficiency effectively. To ensure this real-time performance, we optimized the GNN-tracker architecture by adopting lightweight operations within the GCN and Transformer modules, alongside an efficient graph construction process. These architectural choices, combined with the integration of multi-sensor fusion, graph-based modelling, and Transformer-based feature attention, collectively contribute to its superior tracking performance under diverse and challenging conditions.

The results presented in Table 2 highlight the superior performance of GNN-tracker (fused) in handling various challenging tracking conditions, including OCC, BC, LR, FM, out-of-view (OV), and IPR. The model achieves the highest OCC scores, with 85.3% in optical and 83.2% in thermal, outperforming TransT by 11.8% and 13.5%, respectively. This improvement is attributed to graph-based feature aggregation and the Transformer-based attention mechanism, which effectively reconstruct missing features and preserves identity consistency under OCCs. In terms of BC, GNN-tracker (fused) attains 79.6% accuracy in optical and 77.5% in thermal, significantly surpassing ByteTrack, which achieves only 60.5% and 55.7%, respectively. This demonstrates that multi-sensor fusion helps differentiate UAVs from cluttered backgrounds, particularly in the thermal domain. The model also excels in low-resolution scenarios, achieving 80.2% in optical and 78.9% in thermal, outperforming TransT by 10% and 13.1%, respectively. The Transformer-based feature refinement ensures better localization, whereas other models, particularly SORT and DeepSORT, struggle due to their reliance on high-resolution appearance features. When handling FM, GNN-tracker (fused) records an FM score of 83.4% in optical and 81.0% in thermal, demonstrating strong adaptability to high-speed UAVs. The graph structure propagates temporal dependencies while the Kalman filter refines trajectory estimates, reducing errors in high-speed conditions. In comparison, ByteTrack and TransT lag behind by 17.3% and 12.6%, respectively. OV tracking remains another challenging aspect, but GNN-tracker (fused) achieves 81.1% in optical and 79.4% in thermal, outperforming TransT by 11.7% and 14.3%, respectively. The graph-based representation enhances object association, mitigating identity fragmentation when UAVs reappear after temporary disappearances. Lastly, IPRs alter UAV appearance, often leading to tracking failures, but GNN-tracker (fused) achieves an IPR score of 82.7% in optical and 80.6% in thermal, maintaining identity consistency despite rotations. The Transformer-based feature attention mechanism enables robust spatial representation, reducing misclassification errors under extreme rotations. In contrast, TransT and ByteTrack show moderate performance, struggling with severe rotations due to their less effective feature adaptation mechanisms. Overall, these findings confirm that GNN-tracker (fused) significantly outperforms state-of-the-art models across all tracking challenges. The graph-based modelling, Transformer-enhanced feature extraction, and multi-sensor fusion contribute to its superior robustness under OCCs, motion blur, and low visibility conditions. Notably, the fusion of optical and thermal data enhances tracking accuracy, particularly in scenarios with BC, OCCs, and low-resolution imagery. Compared to existing models, GNN-tracker (fused) consistently surpasses TransT, ByteTrack, and DeepSORT, achieving the highest scores across all attributes. These results underscore the importance of integrating spatial-temporal graph modelling with multi-sensor fusion, enabling the model to adapt to dynamic UAV behaviours more effectively than traditional methods. These findings highlight the strong potential of GNN-tracker for real-world UAV tracking applications, where environmental conditions vary significantly, and tracking robustness is essential.

**Table 2:** Results obtained under different attributes for different methods with optical, thermal, and fused dataset

Tracker	OCC ↑	BC ↑	LR ↑	FM ↑	OV ↑	IPR ↑
SORT (optical)	54.3%	46.2%	49.5%	50.9%	48.7%	51.3%
DeepSORT (optical)	61.8%	53.6%	55.7%	57.4%	55.9%	58.1%
ByteTrack (optical)	68.2%	60.5%	62.4%	66.1%	63.3%	65.7%
TransT (optical)	73.5%	66.8%	70.2%	72.0%	69.4%	71.8%
GNN-tracker (fused)	85.3%	79.6%	80.2%	83.4%	81.1%	82.7%
SORT (thermal)	49.2%	42.7%	45.1%	46.8%	43.5%	44.9%
DeepSORT (thermal)	57.4%	48.9%	52.0%	55.1%	50.8%	52.3%
ByteTrack (thermal)	63.9%	55.7%	59.1%	61.2%	58.4%	60.5%
TransT (thermal)	69.7%	62.9%	65.8%	68.4%	65.1%	67.3%
GNN-tracker (fused)	83.2%	77.5%	78.9%	81.0%	79.4%	80.6%

To justify the high frame rate achieved by the GNN-tracker (58.9 FPS in the fused configuration), we performed a detailed runtime profiling analysis of its main computational modules. The Transformer-based feature extraction accounts for approximately 50% of the inference time, the GCN layers for 30%, and the multi-sensor fusion module for 20%. This distribution confirms that the model is well-balanced in terms of computational load and validates the design choice to integrate lightweight, parallelizable modules. The relatively low overhead of the fusion module also highlights the efficiency of the adaptive weighting mechanism used to combine thermal and optical features.

To support such real-world deployment at scale, it is also crucial to consider the model’s computational behaviour as UAV density increases. The computational complexity of the GNN-tracker increases approximately linearly with the number of UAV detections due to the graph construction and message-passing procedures. By leveraging parallelized Transformer and GCN computations, the system maintains real-time performance for moderate-to-high target densities. However, in extremely dense scenarios, additional computational overhead may slightly impact performance. Future work will focus on further optimizing these steps to enhance scalability on resource-limited platforms.

#### Ablation study and comparative analysis

To evaluate the contribution of each component in GNN-tracker (fused), an ablation study was conducted by systematically removing key modules, including the Transformer module, GCN layers, and multi-sensor fusion. The results, summarized in Table 3, demonstrate the impact of each component on overall tracking performance across different challenging attributes. The study confirms that each module plays a crucial role in improving tracking accuracy and robustness. Removing the Transformer module results in a 7.8% drop in HOTA, revealing that the self-attention mechanism is essential for long-range feature refinement and object re-identification under OCCs and BC. Additionally, the absence of the GCN layer causes a drastic decline in MOTA and IDF1, highlighting the importance of graph-based feature aggregation for maintaining identity consistency and spatial-temporal correlation. This leads to increased fragmentation in UAV trajectories and a higher occurrence of IDSW.

**Table 3:** Ablation study results on optical, thermal, and fused datasets

Model variation	MOTA	IDF1	HOTA	OCC	BC	LR	FM	OV	IPR	FPS
GNN-tracker (w/o Transformer)	81.6%	76.2%	72.3%	72.5%	67.9%	69.0%	70.4%	68.1%	71.3%	47.9
GNN-tracker (w/o GCN)	77.9%	71.4%	68.5%	67.9%	61.5%	65.2%	65.2%	63.7%	67.4%	45.1
GNN-tracker (w/o multi-sensor fusion)	80.2%	73.9%	70.6%	70.1%	65.3%	68.0%	68.0%	66.4%	69.5%	49.6
Full GNN-tracker (optical)	89.1%	84.7%	80.1%	82.4%	76.9%	78.3%	80.7%	78.5%	79.6%	56.8
Full GNN-tracker (thermal)	86.3%	81.2%	78.7%	78.9%	73.3%	74.7%	77.2%	74.6%	76.1%	54.3
Full GNN-tracker (fused)	91.4%	86.8%	82.3%	85.3%	79.6%	80.2%	83.4%	81.1%	82.7%	58.9

The multi-sensor fusion mechanism is particularly significant in scenarios where thermal and optical data complement each other. Without this fusion, MOTA decreases by 8.9%, demonstrating that integrating multi-modal sensor data is crucial for enhancing detection in challenging environments such as low visibility, BC, and extreme lighting conditions. Furthermore, without fusion, the FNR increases, confirming that thermal imaging aids in detecting UAVs that may be obscured in the optical spectrum.

From Table 3, it is evident that the full GNN-tracker (fused) model achieves the highest performance across all evaluation metrics, with a MOTA of 91.4%, HOTA of 82.3%, and an FPS of 58.9. This confirms that the integration of Transformer-based attention, GCN for spatial-temporal modelling, and multi-sensor fusion collectively ensures optimal UAV tracking across diverse operational conditions. Compared to reduced versions of the model, the full version demonstrates superior adaptability to OCCs, FM, and OV scenarios, making it the most robust UAV tracking solution tested in this study.

These consistent improvements across thermal, optical, and fused domains validate the robustness of GNN-tracker in diverse UAV tracking environments.

## Conclusions

In this paper, we proposed GNN-tracker, a novel GNN-based UAV tracking framework that integrates graph-based spatial-temporal modelling, Transformer-based feature extraction, and multi-sensor fusion to enhance tracking accuracy and robustness. The experimental evaluation demonstrated that GNN-tracker (fused) significantly outperforms state-of-the-art methods across multiple tracking benchmarks, particularly in challenging conditions such as OCC, BC, LR, FM, OV scenarios, and IPR.

Our results show that GNN-tracker (fused) achieved a MOTA of 91.4%, a HOTA of 82.3%, and an FPS of 58.9, surpassing TransT by 8.9% in MOTA and 7.7% in HOTA. The integration of multi-sensor fusion proved essential for handling thermal-optical variations, leading to an 8.9% performance gain in MOTA compared to a model without fusion. Additionally, the Transformer-based attention mechanism contributed significantly to tracking robustness, preventing IDSW and improving UAV detection in cluttered environments. The GCN module was shown to be vital for maintaining spatial-temporal correlations, as removing it resulted in an 11.2% drop in MOTA, demonstrating its role in object association accuracy.

With real-time FPS and efficient module design, GNN-tracker is well-suited for deployment in onboard UAV tracking systems under real-world constraints.

A comprehensive ablation study highlighted the critical role of each component, proving that the combination of graph modelling, Transformer feature refinement, and multi-sensor fusion provides an optimal balance between accuracy and computational efficiency. The findings validate GNN-tracker’s effectiveness in real-world UAV tracking applications, where environmental conditions vary significantly, requiring a model capable of adapting dynamically to these challenges.

Despite its strong performance, GNN-tracker still faces certain limitations. Computational complexity remains an area for improvement, and further optimizations could enhance its deployment in resource-constrained environments. Additionally, the model’s adaptability could be further refined by integrating self-supervised learning techniques to reduce dependency on labelled data and enhance generalization across diverse UAV tracking environments.

For future work, we plan to extend GNN-tracker by developing lightweight architectures (e.g., using MobileNet backbones or pruning strategies) for edge-device deployment, ensuring real-time tracking with minimal computational overhead. Although the current experiments are conducted on high-performance GPUs, the architecture is modular and adaptable for embedded UAV platforms such as Jetson Xavier or Nano.

Additionally, we aim to enhance multi-modal fusion strategies by incorporating radar and LiDAR data to improve UAV detection in extreme weather conditions. Overall, GNN-tracker presents a significant advancement in UAV tracking, offering a highly accurate, robust, and efficient framework capable of handling complex real-world scenarios.

## Data Availability

The datasets generated and analysed during this study are not publicly available due to confidentiality restrictions and cannot be obtained from the corresponding author.

## Abbreviations

UAV: Unmanned aerial vehicle
GNN: Graph neural network
MOTA: Multiple object tracking accuracy
HOTA: Higher order tracking accuracy
CNN: Convolutional neural network
GCN: Graph convolutional network
MOT: Multiple object tracking
FNR: False negative rate
FM: Fast motion
OCC: Occlusion
BC: Background clutter
IPR: In-plane rotation
LR: Low resolution
IDF1: Identification F1
FPR: False positive rate
IDSW: Identity switches
FPS: Frames per second
OV: Out-of-view
